# Therapeutics

**Published:** 1878-05

**Authors:** 


### Therapeutics.

Vaseline and Salicylic Acid in Obstetrics.—Dr. Dubois. (*Med. Record.*)

Vaseline is a hydrocarbon, made from petroleum by simple evaporation and clarification. It is very cheap, being worth only some forty to fifty cents a pound. It has no taste or smell. Its rôle as a protective against the action of the air is extensive, as in burns, excoriations, etc. It is one of the best of lubricants. Its use is simple, and especially in complicated labors is thus very advantageous. Internally, it seems to relieve irritation of the mucous membrane, and, when taken up by the system, though it undergoes no proper digestion, to act much in the same way as cod-liver oil. As a vehicle for more active agents, it is more generally useful than any other oil-like compound. Salicylic acid has of late come into vogue, and is now used for a great variety of purposes—principally as an antiseptic, to reduce the heat of the body, and in diseases in which there is a morbid material in the blood, as in rheumatism, and gout, etc. It is not expensive, costing from thirty to forty cents an ounce. I have tried several samples of different manufacture, and find that of Rosengarten, of Philadelphia, by far the best, while the German article that I have used has proved caustic and utterly unfit for many purposes. The American acid is in silky, white crystals, like quinine, has no caustic taste, and, mixed with vaseline, makes a homogenous ointment. The German is amorphous, looks like chalk, has a slight pinkish color and caustic taste, and, mixed with vaseline, makes a lumpy, irritating ointment, unfit for use.

It has been my practice for some time back to use vaseline, with a grain or more of salicylic acid to the ounce, and scented with a drop of ottar of roses, in all vaginal examinations, instead of oil or soap. I believe I thereby more certainly avoid carrying infection from case to case that I should otherwise do. In first confinements it may be used in the first state of the labor, so soon as the woman takes to bed. I make use of a glass syringe, an inch in diameter, without a nozzle. With an instrument of this kind an ounce or more of the semi-solid vaseline can be introduced up to the os, where it remains at the temperature of the body, in a semi-solid state. I use it in this way as a simple lubricant, and without the addition of the acid. If desirable, in certain cases, it can be combined with the extract of belladonna, and, after the labor is completed, with the extract of ergot, or, in case of hemorrhage, with the liq. ferri persulphatis, with all of which it mixes well. If it is desired to introduce it into the uterus, it can be rendered fluid by putting the bottle containing it into water of a temperature of 100° F., when it can be used with the ordinary uterine syringe. In the course of a labor I use three to six ounces, with the effect, as I claim, of shortening the first stage of labor, and rendering the parts, especially in first labors, easily dilatable in the second stage, while, after the placenta is delivered, a small quantity of the vaseline, with the acid added, disinfects the discharges, and does much, it seems to me, to prevent purulent absorption. Indeed, if puerperal fever was prevalent, I should not hesitate to introduce it freely into the uterus immediately after confinement. To illustrate the healing qualities of this combination, I some time ago had an extensive rupture of the perineum in a primipara, due to an unusually large child and an unyielding perineum. I passed two pins through the lips of the wound, and a figure-of-eight around each, and directed the patient to introduce a little of the vaseline ointment two or three times a day on her finger. On the third day after, when I next saw her, on removing the pins I found the wound entirely healed. My cases are not sufficient to base positive conclusions on, but I am inclined to think that an hour or more can be saved in an ordinary labor by the use of the vaseline, and that the second stage will go on easier owing to a more thorough relaxation of the soft parts, and to the avoidance of unnecessary friction; and that its use, with the acid after labor, will do much to prevent puerperal absorption, and, in any event, will conduce to the comfort of the patient. In dilating the os with the sponge tent, I find that by coating it with the vaseline and the acid (ten grains to the ounce), I can more readily introduce it, the tent not expanding at first, owing to the coating of vaseline; but, if held for a moment or two in place, it will remain without danger of its coming away, and will expand to the same limits that it would have done without the coating of vaseline, as can easily be proved by putting two tents in water, one coated and the other not. In erosions of the os, after the engorgement of the parts is removed by glycerine pads, the vaseline and acid ointment, applied on cotton-wool, will do much to effect a speedy cure, especially if alternated with the glycerine. There is one use for this ointment that I have not fully worked out. Physicians are frequently applied to, to produce abortion. Recently, on the same day, two women came to me; the reason assigned in the one case was that the husband was syphilitic; in the other that pregnancy brought on violent attacks of spasmodic asthma. Of course I explained that the child had rights as well as the mother, but it was all that I could do to prevent one of these cases from going to a professed abortionist. In some cases of this kind prevention is better than cure, and I am inclined to think, from some experiments, that vaseline, charged with four to five grains of salicylic acid, will destroy spermatozoa, without injury to the uterus or vagina.

In conclusion, there are a number of uses for vaseline in the lying-in room and nursery. I make no claim to its being a "cure-all," but it is a great convenience, and its "role" is extensive. The ointment makes a good dressing for the umbilical cord. Vaseline answers better than oil or soap to remove the cerumen from the newly-born infant. Mixed with an equal weight of honey and ten grains of borax or of chlorate of potassa to the ounce, it answers an excellent purpose in case of thrush.

The ointment alone, or mixed with ten grains of quinine to the ounce, quickly removes the small worms that frequently infest the anus of young children. In the excoriations of infants it effects rapid healing. In the not uncommon sore eyes of the first few days of life, the vaseline alone introduced within the eyelids, effects a cure in a day or two. Again, in the "snuffles" of the old women, which, by preventing nursing, frequently seriously affect the health of the infant, it, when introduced into the nostrils with a camel's-hair pencil, answers better than anything I have as yet tried, especially if the head is kept warm with a flannel cap. There are many other uses for vaseline, alone or combined with varying proportions of salicylic acid, that the experience of the physician will readily suggest to him in this connection.

---

At the examination of the Illinois State Board of Health, held in this city on March 21st, out of a class of sixteen, only four passed. On April 11th, at Centralia, twenty-six received certificates out of forty-one applicants. Of these, nineteen had failed before, some two, and others three times, their examinations extending over a period of five months. Their perseverance was certainly commendable, and their final success was at least some evidence that they made good use of their time, prompted by the persuasive influence of necessity. Until recently no attention has been paid to midwives. The same rules will be applied to them as to practitioners.

Mr. Wordsworth exhibited to the Medical Society of London, last January, six cases of congenital displacement of both lenses, in one family—mother, two sons and their three children. They state that their ancestors were troubled similarly; thus making, if their statement be true, *ten* cases in *five* generations. They are all myopic.—(*Dublin Jour. of Med. Sci.*, March, 1878.)

